# Formally exact simulations of mesoscale exciton dynamics in molecular materials†

**DOI:** 10.1039/d1sc01448j

**Published:** 2021-05-31

**Authors:** Leonel Varvelo, Jacob K. Lynd, Doran I. G. Bennett

**Affiliations:** Department of Chemistry, Southern Methodist University PO Box 750314 Dallas TX USA doranb@smu.edu

## Abstract

Excited state carriers, such as excitons, can diffuse on the 100 nm to micron length scale in molecular materials but only delocalize over short length scales due to coupling between electronic and vibrational degrees-of-freedom. Here, we leverage the locality of excitons to adaptively solve the hierarchy of pure states equations (HOPS). We demonstrate that our adaptive HOPS (adHOPS) methodology provides a formally exact and size-invariant (*i.e.*, 
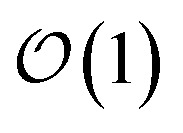
) scaling algorithm for simulating mesoscale quantum dynamics. Finally, we provide proof-of-principle calculations for exciton diffusion on linear chains containing up to 1000 molecules.

## Abstract 2

The adaptive hierarchy of pure states (adHOPS) algorithm leverages the locality of excitons in molecular materials to perform formally-exact simulations with size-invariant (*i.e.*, ) scaling, enabling efficient simulations of mesoscale exciton dynamics.

## Graphical abstract

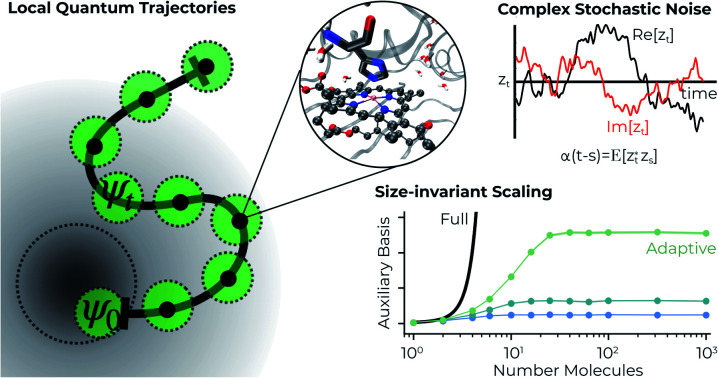

## Introduction

New molecular materials, particularly organic semiconductors, offer remarkable and tunable functionality for photonic, opto-electronic, and light harvesting applications. The photophysical properties of molecular materials arise from diffusion of excited-state carriers (*e.g.*, electronic excitations, called ‘excitons’) across the 10 nm to 1 µm length scale. These mesoscale exciton dynamics are sensitive to both the molecular properties of the material building blocks and structural heterogeneities, which include everything from point defects to grain boundaries. Traditional bulk spectroscopies provide only indirect evidence for the essential role of structural heterogeneity in exciton transport. The recent development of spatially-resolved non-linear spectroscopy provides a remarkable new lens by which to study exciton dynamics in heterogeneous materials.^1,2^ Interpreting spatially-resolved spectroscopic signals, however, remains challenging due to the absence of corresponding simulations.

Simulating exciton transport dynamics in heterogeneous materials on the 10 nm to 1 µm length scale (*i.e.*, the mesoscale) remains an outstanding theoretical challenge. Organic semiconductors often combine close intermolecular packing with correspondingly large coupling between electronic states (*V*) on adjacent molecules and large intramolecular electron-vibrational coupling (*λ*).^3^ Perturbative equations-of-motion, such as Förster theory, can be convenient for simulating large aggregates, but are not applicable when *V* and *λ* are comparable in magnitude. Similarly, in the absence of a clear separation of timescales between vibrational and electronic degrees-of-freedom, Markovian equations-of-motion, such as Redfield theory,^4^ struggle to capture the rich dynamics of excitation transport. There are a variety of non-perturbative, non-Markovian equations-of-motion, such as multi-layer multi-configuration time-dependent Hartree (ML-MCTDH),^5,6^ time-evolving density operator with orthogonal polynomials (TEDOPA),^7^ hierarchically-coupled equations-of-motion (HEOM),^8^ and quasi-adiabatic path integrals (QUAPI).^9^ All of these techniques, however, share an exponential scaling of computational complexity with the number of molecules. While efficient and parallelized implementations of formally exact methods have been developed – for example, distributed memory HEOM^10,11^ – the exponential scaling severely limits even high-performance simulations of molecular aggregates.

Recently, there have been a few notable developments towards highly-scalable equations-of-motion for exciton dynamics. Modular path integrals^12,13^ provide a dramatic reduction in computational cost of QUAPI, but retain an overall linear scaling with the number of molecules and are most efficient when molecules exhibit only nearest-neighbor coupling. Dissipation-assisted matrix product factorization (DAMPF)^14^ extends TEDOPA to efficiently describe large numbers of vibrational degrees-of-freedom (>10) on each molecule, but it maintains between a quadratic and cubic scaling with the number of molecules. For both modular path integrals and DAMPF, the residual scaling makes it challenging to apply these methods to mesoscale calculations containing thousands to millions of molecules. Indeed, any density matrix approach will suffer from residual scaling with system size at long times due to the spread of ensemble population density across molecules.

Stochastic simulations, which decompose the ensemble into a collection of excited trajectories, can enable calculations on arbitrarily large molecular aggregates, even at long time. Delocalized kinetic Monte Carlo^15^ and the kinetic Monte Carlo version of generalized Förster theory^16^ are stochastic approaches that calculate the rate of transport between clusters of strongly interacting molecules and can be readily extended to mesoscale calculations. Both of these methods, however, use a perturbative approximation to partition state space and calculate rates between adjacent spatial regions. The development of a non-perturbative, non-Markovian approach for mesoscale simulations would provide an important benchmark for new equations-of-motion and could offer insight into processes with debated mechanisms, such as charge separation in organic photovoltaic materials.^15,17–19^

Here, we present a non-perturbative, non-Markovian, and arbitrarily scalable stochastic method for simulating exciton transport. First, we introduce the Hamiltonian considered and our base equation-of-motion, the hierarchy of pure states (HOPS).^20^ Next, we discuss locality in HOPS calculations and present an algorithm for constructing an adaptive basis. Finally, we present proof-of-concept calculations using the adaptive HOPS (adHOPS) equation-of-motion that demonstrate both its accuracy and size-invariant (*i.e.*, 
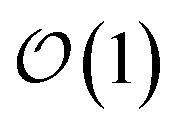
) scaling for large molecular aggregates.

## Preliminaries

### Hamiltonian

We divide the exciton Hamiltonian into three parts1
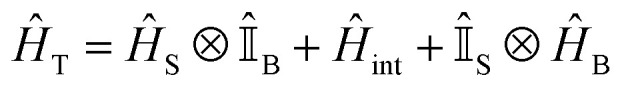
where 
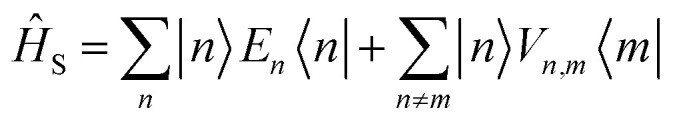
 describes the electronic system and 
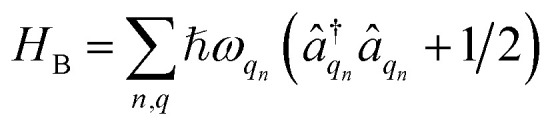
 represents the thermal environment arising from molecular vibrations. The influence of coupling between the electronic system and vibrational ‘bath’ 
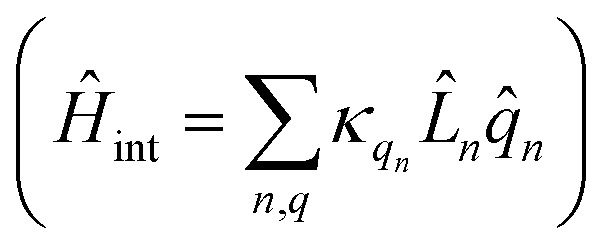
 can be described in terms of the system-bath coupling operators 
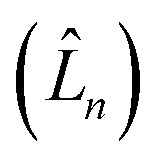
 and the two-point correlation functions2
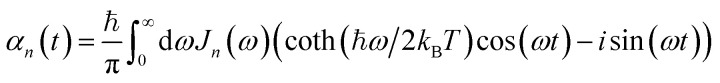
where *T* is the temperature and 
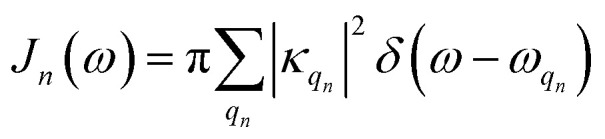
 is the spectral density. In the following, we assume that each pigment has an independent thermal environment that drives fluctuations in excitation energy. In other words, we assume that the system-bath coupling operator is a site-projection operator 
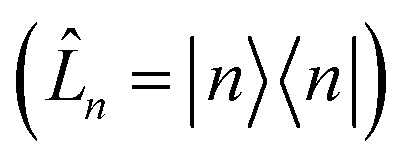
. We describe the thermal environment of each pigment by a Drude–Lorentz spectral density3
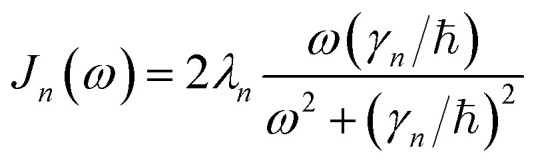
which, at high temperature (*γ*_*n*_/*k*_B_*T* < 1), allows for a convenient exponential decomposition of the correlation function

4 *α*_*n*_(*t*) = *g*_*n*_e^−*γ*_*n*_*t*/ℏ^

where *g*_*n*_ = 2*λ*_*n*_*k*_B_*T* − *iλ*_*n*_*γ*_*n*_. In the following we use *λ*_*n*_ = *γ*_*n*_ = 50 cm^−1^, *V* = 25–250 cm^−1^ and *T* = 295 K, which are comparable to the parameters used for many simulations of photosynthetic pigment protein complexes and fall into the broad intermediate regime where perturbative approximations break down.^21^

### Hierarchy of pure states (HOPS)

The non-Markovian quantum state diffusion (NMQSD) equation^22^ decomposes the time-evolution of the reduced density matrix for the system degrees-of-freedom into an ensemble average over stochastic pure states indexed by a complex stochastic processes *z*_*n*,*t*_5
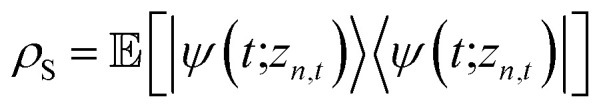
where 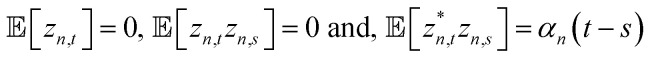. The equation-of-motion for the independent stochastic trajectories is6
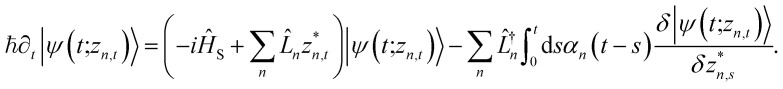


The NMQSD equation is formally exact and is equivalent to solving Feynman path integrals with the Feynman–Vernon influence functional,^22^ but the functional derivative in the last term makes direct solution of the stochastic trajectories impractical except in special cases.

The hierarchy of pure states (HOPS) equations provide a numerically tractable version of NMQSD by rewriting the functional derivative as a set of coupled differential equations.^20^ Briefly, the sum of integrals over a functional derivative in the final term of the NMQSD equation is defined as a sum of first order auxiliary wave functions:7
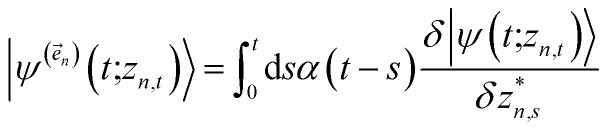
giving8
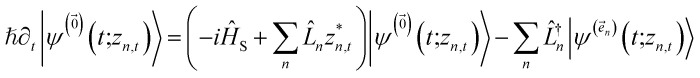
where we have now introduced a vector label into the equations to index the different components. The physical wave function is given by 
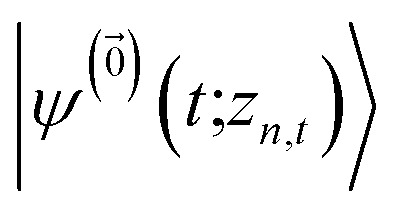
. The first order auxiliaries are indexed by unit vectors with non-zero index at their *n*^th^ element 
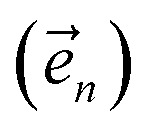
. When the correlation function *α*_*n*_(*t*) is written as an exponential (or sum of exponentials), the time-evolution of the first order auxiliary wave functions 
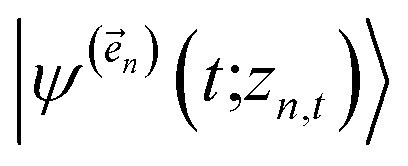
 introduces the second-order auxiliary wave functions 
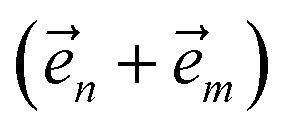
, and so on, *ad infinitum*. The resulting general expression, called the ‘linear HOPS equation,’ is9
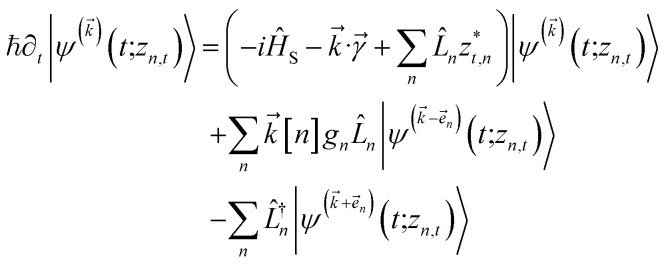
where we have introduced a general vector 
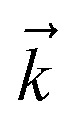
 to index auxiliary wave functions, 
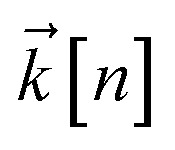
 is the *n*^th^ element of the index vector, 
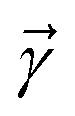
 is the vector of correlation function exponents (*γ*_*n*_), and terms involving any auxiliary wave function with an indexing vector containing a negative element are always zero. The linear HOPS equation maintains the normalization of the system reduced density matrix within the ensemble average, but the physical wave function is not normalized in individual trajectories. Instead, for long trajectories, most realizations have 
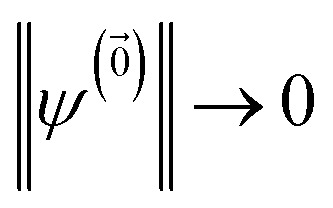
 and an infinitesimal subset have physical wave functions with diverging norms.^20^ As a result, linear HOPS calculations exhibit slow convergence with respect to the size of the ensemble.

We can improve convergence with ensemble size by using the non-linear HOPS equation, which describes the time-evolution of a normalizable stochastic wave function. We rewrite the reduced system density matrix (eqn (5)) in terms of a normalized wave function and the norm contribution10
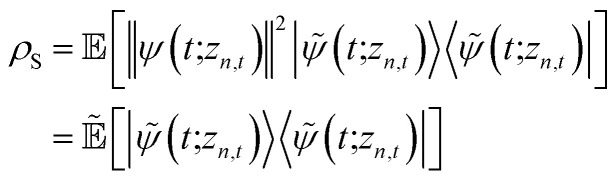
where 
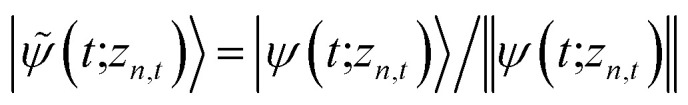
. The norm in the first expression can be interpreted as a weighting factor for a new ensemble average. Using a Girsanov transform, we can solve for the corresponding equation-of-motion for |*ψ*(*t*;*z*_*n*,*t*_)〉,^23^ which gives the non-linear HOPS equation^20^11
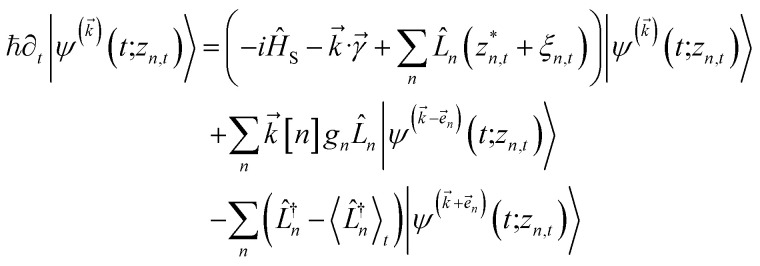
where12
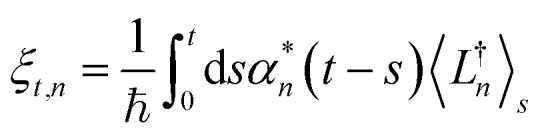
is a memory term that causes a drift in the effective noise, and13
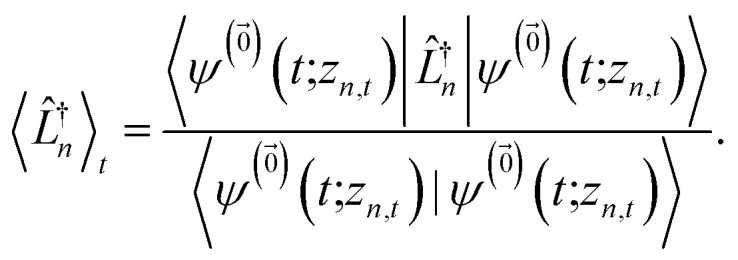


We note that the non-linear HOPS equation ensures that the contribution of each wave function is normalized in the reduced density matrix, but it time-evolves the non-normalized physical wave function. In the following, we will drop the explicit *z*_*n*,*t*_ dependence from the wave function for simplicity 
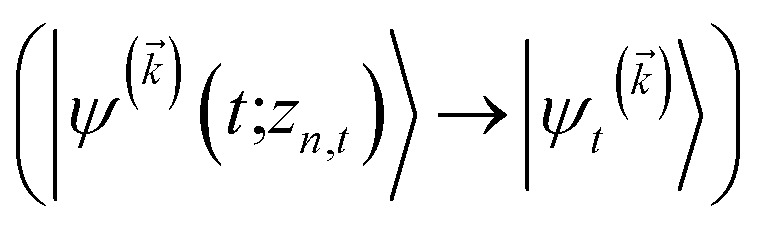
.

The HOPS equations are a numerically convenient, formally exact expression for exciton dynamics in small molecular aggregates. Moreover, the calculations are ‘embarrassingly’ (also called ‘perfectly’) parallel^24^ due to the independence of individual trajectories, and, as a result, HOPS ensembles can be computed using thousands of CPUs simultaneously without loss of efficiency. The application of HOPS to large molecular aggregates, however, is limited by the scaling of the HOPS basis with the number of molecules. It is convenient to think of HOPS calculations as depending on two basis sets: the state basis 
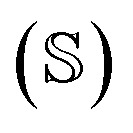
 and the auxiliary basis 
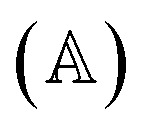
. The complete state basis is a finite set of vectors that span the Hilbert space of the system, while the complete auxiliary basis is composed of an infinite set of auxiliary wave functions indexed by vectors 
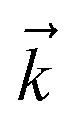
. To construct a finite auxiliary basis, the infinite hierarchy must be truncated. Here, we employ the common triangular truncation condition which limits the auxiliary basis to those wave functions with index vectors 
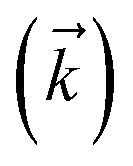
 that have a sum of elements less than a preselected bound *k*_max_
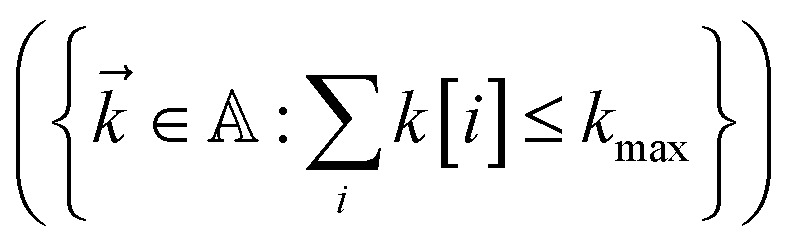
. If we assume one independent environment per state, then the number of auxiliary wave functions included in the triangular truncation scales as 
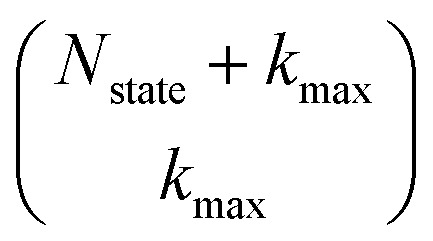
 which gives an overall 
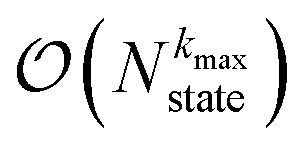
 scaling for large aggregates. While convergence as a function of *k*_max_ is guaranteed, the requisite number of auxiliary wave functions is often prohibitive.

### Short-time correction and Markovian modes

The Drude–Lorentz correlation function given in eqn (4) has a discontinuity at *t* = 0 arising from the symmetry condition14
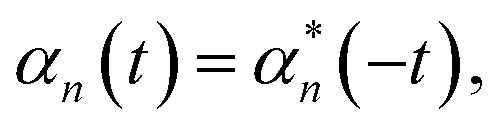
and can be more completely written as^25^

15 *α*_dl,*n*_(*t*) = (Re[*g*_*n*_] + *i*sgn(*t*)Im[*g*_*n*_])e^−*γ*_*n*_|*t*|/ℏ^

The discontinuity in the correlation function introduces a numerically inconvenient infinitely high-frequency component to the stochastic noise trajectories *z*_*n*,*t*_.

We ameliorate this problem by redefining the positive-time correlation function in terms of two continuous exponential functions *α*(*t*) = *α*_0,*n*_(*t*) + *α*_mark,*n*_(*t*) where

16 *α*_0,*n*_(*t*) = *g*_*n*_e^−*γ*_*n*_|*t*|^^/^^ℏ^

and

17 *α*_mark,*n*_(*t*) = −*i*Im[*g*_*n*_]e^−*γ*_mark_|*t*|^^/^^ℏ^.

The definition of *α*_mark,*n*_(*t*) ensures the imaginary component of the total correlation is 0 when *t* = 0, and it also provides for a smooth transition back to the naive correlation function *α*_0,*n*_(*t*) on a finite timescale given by ℏ/*γ*_mark_. Except where otherwise noted, *γ*_mark_ = 500 cm^−1^ for all calculations presented in the main text, because this was sufficiently fast to ensure the Markovian timescale had no influence on the calculated dynamics.

Due to the extremely rapid timescale on which *α*_mark,*n*_(*t*) decays, this mode is Markovian, and high-lying contributions to the hierarchy can be neglected. In the following, we will only include the first order terms associated with these Markovian modes and neglect these terms in our discussion of the auxiliary wave functions forming the hierarchy. This can be viewed as a smoothing of the noise trajectories (*z*_*n*,*t*_) on timescales fast compared to all other dynamics.

This problem can be avoided entirely by using a different spectral density which more naturally accounts for the short-time imaginary component of the correlation function: for example, the recently reported alternative to the Drude–Lorentz oscillator with improved low-temperature behavior.^26^

### Adaptive HOPS (adHOPS)

Within the quantum state diffusion formalism, stochastic wave functions localize in the presence of thermal environments.^27–29^ At a single time point, the pure state can be interpreted as a system wave function conditioned on a measurement of all the environmental degrees-of-freedom.^30^ The delocalization extent of exciton wave functions within the ensemble of pure states is suppressed by the dynamic localization induced by coupling to the vibrational degrees-of-freedom.^31,32^ Previously, Markovian quantum state diffusion calculations have leveraged the locality of the exciton to reduce computational complexity using both a moving basis^27,33^ and an adaptive basis.^34^ Both of these approaches, however, require the conservation of probability, which is violated in the HOPS equations because amplitude in the auxiliary wave functions can be created and destroyed.

Here, we develop an adaptive solution to the HOPS equation-of-motion that achieves size-invariant computational scaling (*i.e.*, 
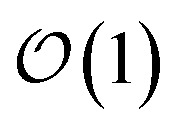
 scaling) for calculations of large molecular aggregates. We first establish a normalized non-linear HOPS equation, which ensures that the magnitude of derivative terms does not diverge with increasing depth of the hierarchy. We then illustrate how locality appears within the hierarchy of auxiliary wave functions, with a particular emphasis on the connection between locality and the flux between neighboring auxiliary wave functions. Finally, we present an adaptive algorithm for the normalized non-linear HOPS equation that satisfies a user-selected bound on the absolute derivative error.

### Normalization of HOPS

To ensure that the magnitude of the derivative elements for auxiliary wave functions have a consistent absolute scale across the hierarchy, we: (1) enforce normalization of the physical wave function in the time-evolution equation and (2) redefine the auxiliary wave function coefficients.

To enforce the normalization of the physical wave function, we rewrite the non-linear HOPS equation in terms of a normalized physical wave function. Starting with eqn (11), dividing all wave functions by the norm of the physical wave function, taking the derivative, and expanding terms gives18
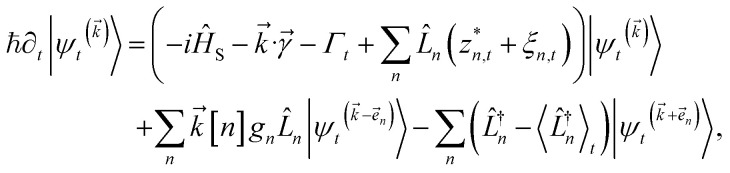
where19
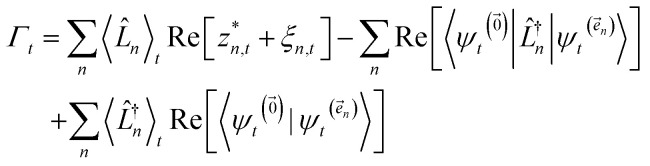
is the normalization correction factor.

**Fig. 1:**
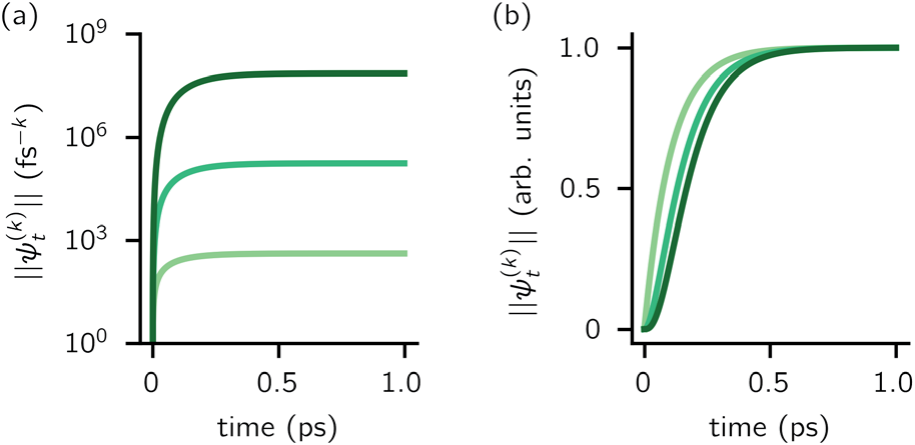
Magnitude of the first three auxiliaries in a single-mode HOPS calculation. (a) The magnitude of the auxiliaries calculated using the non-linear HOPS equation (darker lines correspond to higher *k* values). (b) The magnitude of the auxiliaries calculated using the non-linear HOPS equation with the *k*-dependent prefactor. Parameters: *λ* = *γ* = 50 cm^−1^, *T* = 295 K, and *k*_max_ = 10. No Markovian mode was included in this calculation.

In the non-linear HOPS equation, the magnitude of the auxiliary wave functions grows with increasing auxiliary index. The basic HOPS terminator for a hierarchy with a single thermal environment20
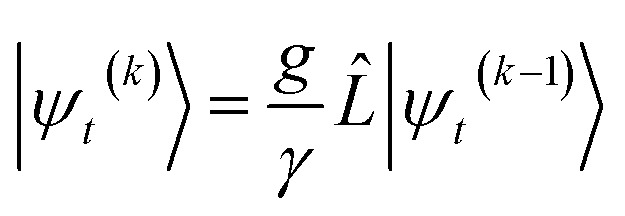
can be derived from the integral form of the HOPS equation by considering the limit where the auxiliary damping is much faster than any system timescale (*kγ*/ℏ ≫ *ω*_sys_).^20^ When this terminator is used for the first-order auxiliaries the resulting HOPS equation is equivalent to the standard Markovian quantum state diffusion equation. Fig. 1a shows the norm of the first three auxiliary wave functions for a single trajectory with a hierarchy consisting of one Drude–Lorentz oscillator. The magnitude 
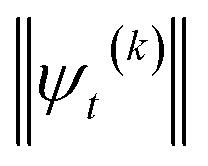
 increases with increasing auxiliary index which, given *g*/*γ* > 1, is consistent with the terminator condition. For a single mode hierarchy, we can ensure the norm of the auxiliary wave functions does not diverge by introducing a new *k*-dependent prefactor for each wave function (*γ*/*g*)^*k*^, as shown in Fig. 1b. In the multimode case, we extend the definition of the prefactor to 
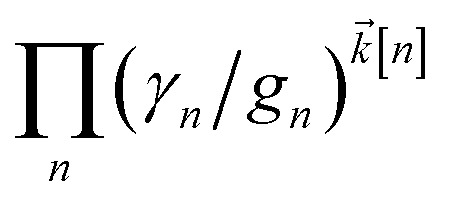
 which ensures that the auxiliary wave functions that define the edges of the hierarchy (only one non-zero mode) do not diverge with increasing hierarchy depth. Rewriting the non-linear HOPS equation to account for this additional prefactor leads to the normalized non-linear HOPS equation21
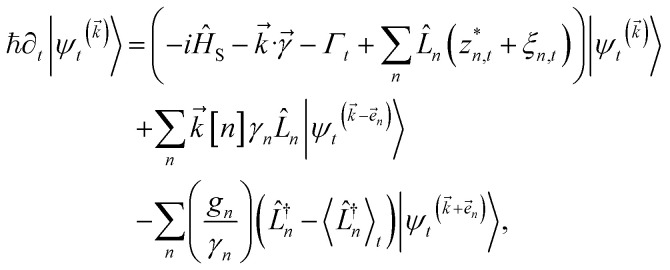
where22
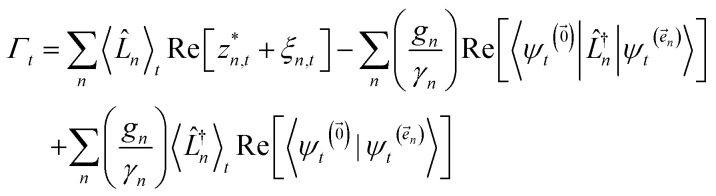
ensures normalization of the physical wave function.

### Locality of HOPS

To construct an adaptive approach to solving the HOPS equations, we must first address the question: how and to what extent does the locality expected in the quantum state diffusion formalism appear in HOPS?

**Fig. 2:**
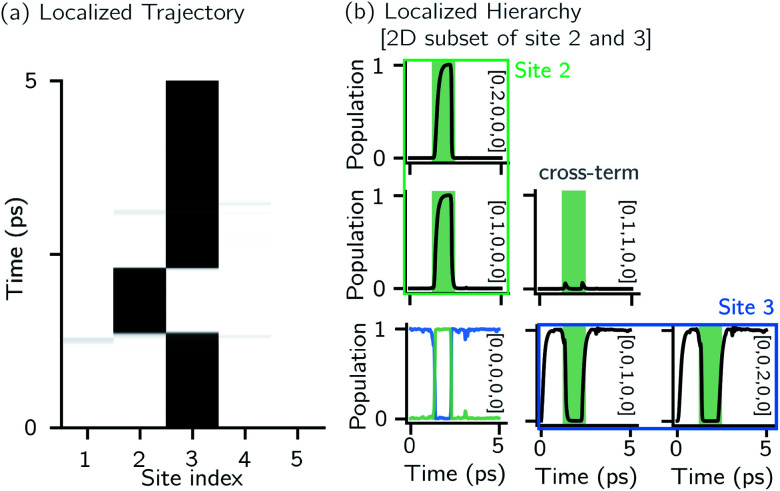
Localization in a single HOPS trajectory. (a) Contour map of site populations in the physical wave function (darker is more populated). (b) Norm-squared of auxiliary wave functions for a two-dimensional subset of the hierarchy associated with site 2 (column) and 3 (row). Panels are labelled by their index vector 
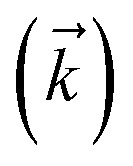
. The shaded region represents the time-period when site 2 is occupied. The physical wave function 
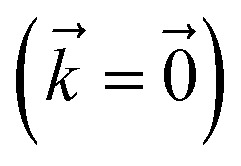
 shows the populations of site 2 and 3 as green and blue lines, respectively. Parameters: *V* = 10 cm^−1^, *λ* = *γ* = 50 cm^−1^, *T* = 295 K, and *k*_max_ = 10.

Fig. 2 shows that in HOPS calculations localization in the physical wave function induces localization in the hierarchy. By ‘localization in the hierarchy,’ we specifically refer to clustering of amplitude in a small set of auxiliary wave functions in a way that depends on the position of the excitation in the physical wave function. In Fig. 2a, an excitation begins on the middle site of a five pigment chain and then jumps between site 3 and site 2. Fig. 2b plots the norm of auxiliary wave functions associated with site 2 and site 3; each plot is labeled by an auxiliary vector index 
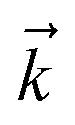
. For example, [0,1,0,0,0] (Fig. 2b, first column) is the first order auxiliary wave function associated with the thermal environment on the second site. The occupation of the auxiliary wave functions (black lines, Fig. 2b) track with the population of the physical wave function – *i.e.*, the auxiliary wave functions associated with the thermal environment of site 2 (first column Fig. 2b) are only occupied when site 2 is occupied in the physical wave function (shaded region).

The locality in the HOPS hierarchy can be understood in terms of the balance of flux terms in the normalized non-linear HOPS equation. First, every auxiliary wave function is damped (first line of eqn (21), 
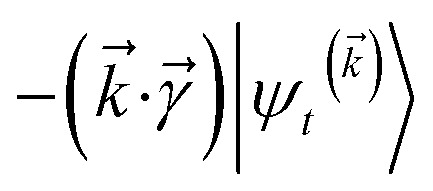
) and, therefore, has zero amplitude without a continuous source term. The fundamental source term is the physical wave function which is the lowest order of the hierarchy. The flux of amplitude towards higher-lying auxiliary wave functions arises from the second line of eqn (21)
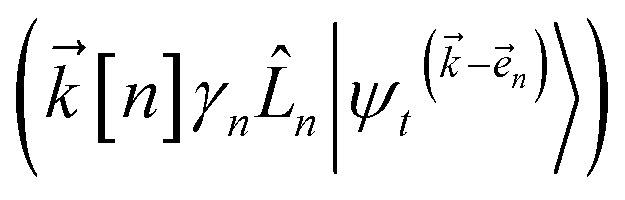
. For system-bath coupling operators that are site projection operators 
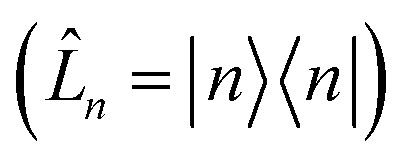
, the flux towards higher-lying auxiliaries only arises when there is amplitude on the associated site of the lower auxiliary wave function. Moreover, the auxiliary wave functions are localized by the same dynamics that localize the physical wave function. As a result, the localized auxiliary wave functions only contribute amplitude to higher-lying auxiliary wave functions with an index that differs by 
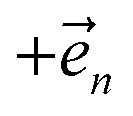
 in a site (*n*) with non-zero amplitude. Thus, the locality of the physical wave function results in preferential population of specific auxiliary wave functions.

### Adaptive algorithm

We have developed an adaptive algorithm for time-evolving the HOPS equations (adaptive HOPS, adHOPS) that leverages locality by constructing a reduced basis set at each time point. We establish the essential basis set elements at each time point (*t*) by ensuring that the error in the time-derivative introduced by the truncated auxiliary 
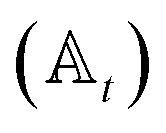
 and state 
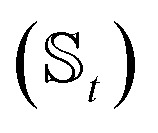
 basis is below a given threshold (*δ*). We define the derivative error in terms of Euclidean distance between the true derivative vector and the effective derivative vector constructed using the adaptive basis. The key equations (given in the ESI†) provide an upper bound on the derivative error squared and are derived by considering all possible flux contributions in the normalized non-linear HOPS equation (eqn (21)), excluding higher order effects introduced through the normalization correction (*Γ*_*t*_). Because auxiliary wave functions share only nearest neighbor connections 
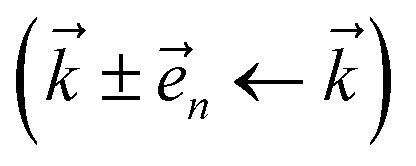
 and the Hamiltonian for a molecular aggregate supports electronic couplings over a finite spatial extent, the adaptive basis can be constructed with 
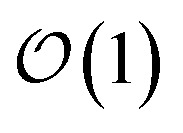
 scaling. The result is a calculation where, in addition to a trajectory of the wave functions, we construct an adaptive basis-set trajectory 
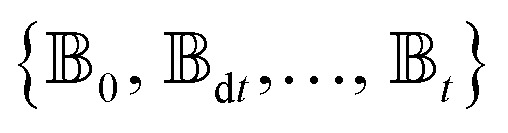
 where 
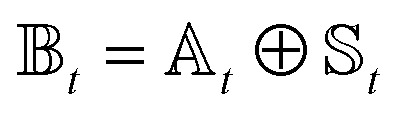
.

Our adHOPS algorithm neither assumes nor imposes locality. Rather, the adaptive basis takes advantage of whatever locality arises during the simulation. If the full hierarchy is required to satisfy the derivative error bound, adHOPS smoothly reverts to a HOPS calculation. As a result, adHOPS remains formally exact – the adaptive basis represents a time-dependent truncation of hierarchy elements, and *δ*, like *k*_max_, is a convergence parameter.

We note that the current adHOPS algorithm makes use of two approximations: first, the spectral density is assumed to be over-damped (*e.g.*, Drude–Lorentz), which allows for a consistent normalization of the hierarchy elements. Second, the system-bath coupling operator is assumed to be a site-projection operator (*L̂*_*n*_ = |*n*〉〈*n*|); in other words, we assume that each molecule has an independent vibrational environment.

## Results and discussion

**Fig. 3:**
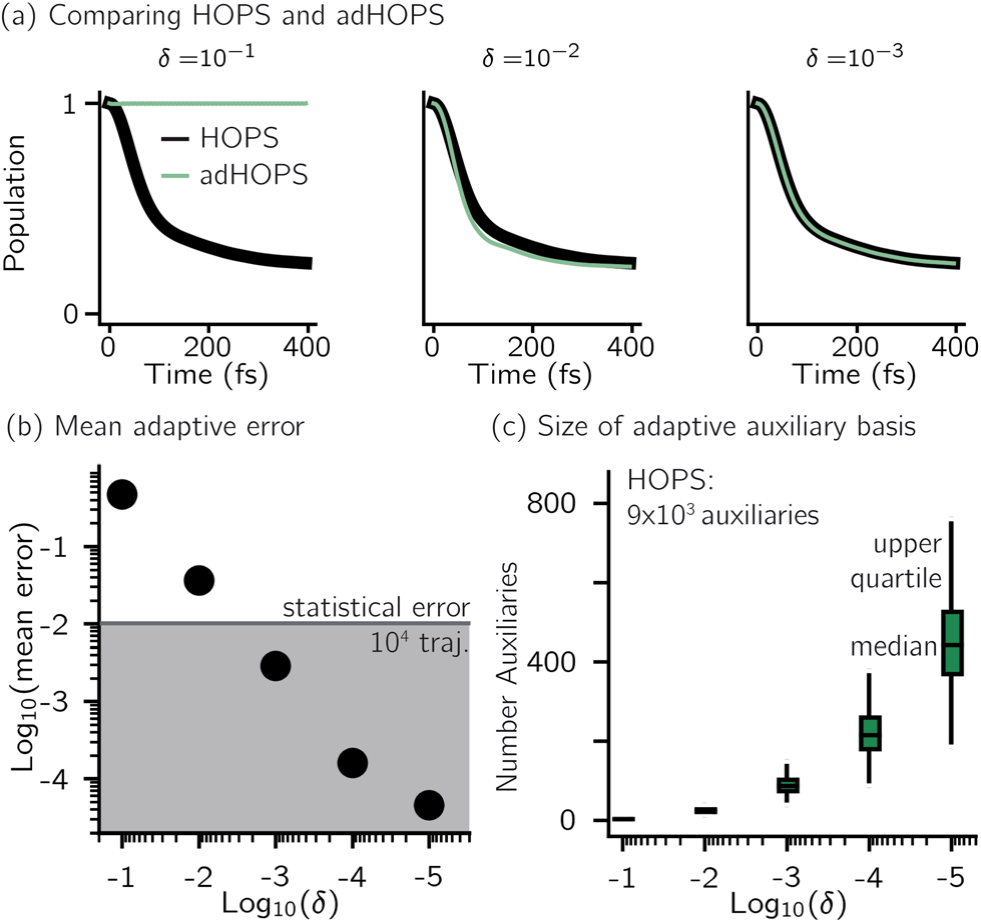
Comparing HOPS and adHOPS for a five-site linear chain. (a) Site 3 population dynamics for HOPS (black line) and adHOPS (green line). (b) Mean adaptive error as a function of *δ*. The grey region represents error beneath the statistical error for a 10^4^ trajectory ensemble. (c) Ensemble distribution of the size of the adaptive auxiliary basis as a function of *δ*. Parameters: *V* = 50 cm^−1^, *λ* = *γ* = 50 cm^−1^, *T* = 295 K, *k*_max_ = 10, and *N*_traj_ = 10^4^.

For a five-site linear chain, adHOPS calculations converge rapidly with respect to the derivative error bound and require only a small fraction of the full HOPS basis. Fig. 3a shows the comparison between full (black line) and adaptive (green line) HOPS population dynamics of the initially excited pigment (site 3). For *δ* = 10^−1^, the adaptive basis set is so small that the calculation shows no excitation transport. Smaller values of *δ* improve the description, and by *δ* = 10^−3^ the mean error is less than 10^−2^. Fig. 3b shows the mean adaptive error as a function of *δ*. In the grey region the adaptive error is smaller than the statistical error associated with the 10^4^ trajectory ensemble. We measure the size of the auxiliary basis for a single trajectory by the average number of auxiliary wave functions required across time points. Fig. 3c plots the ensemble distribution of the auxiliary basis size as a function of *δ*. For *δ* = 10^−3^, most adHOPS trajectories require 10^2^ auxiliaries on average, or approximately 1% of the 9 × 10^3^ auxiliaries required for the corresponding HOPS calculation. Improving the accuracy of the calculation by decreasing *δ* two orders of magnitude only requires about four times as many auxiliaries. The other kinds of error that arise in HOPS simulations, including statistical error from a finite number of trajectories and hierarchy error from the finite *k*_max_ value, are reported in the ESI.†

**Fig. 4:**
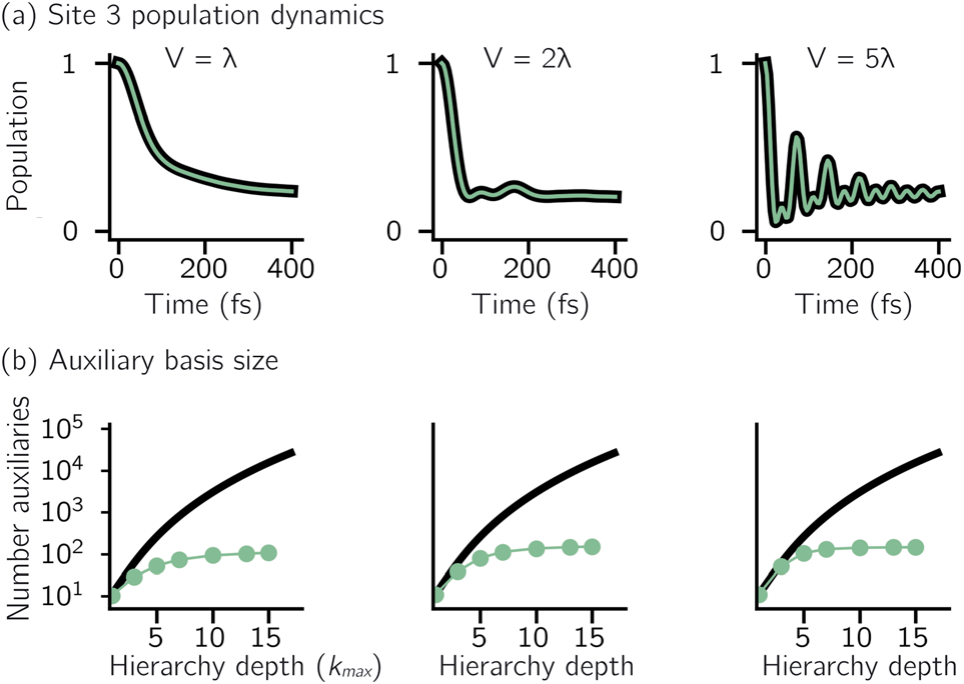
Comparing dynamics and auxiliary basis size as a function electronic coupling (*V*) for the full (black) and adaptive (green) HOPS calculations. (a) Site 3 population dynamics when *k*_max_ = 10. (b) Size of the auxiliary basis as a function of maximum hierarchy depth (*k*_max_). Other parameters: *λ* = *γ* = 50 cm^−1^, *T* = 295 K, *δ* = 10^−3^, and *N*_traj_ = 10^4^. For *V* = 250 cm^−1^, *γ*_mark_ = 1000 cm^−1^, all others used *γ*_mark_ = 500 cm^−1^.

One persistent challenge for numerical implementations of formally exact methods is demonstrating the calculations are converged to the exact answer. In hierarchical methods, calculations must be converged with respect to the auxiliary basis which is defined in the triangular truncation condition by the maximum hierarchy level considered (*k*_max_). In HOPS, the criterion for convergence is that *k*_max_*γ*/ℏ ≫ *ω*_s_, where *ω*_s_ is the characteristic frequency of the system.^20^ Because the full auxiliary basis scales as 
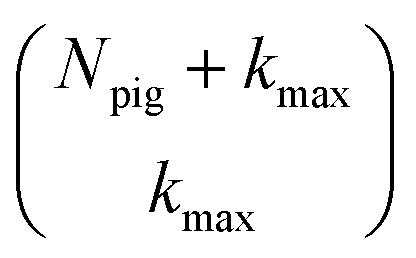
, it is often impractical to systematically check convergence for sufficiently large values of *k*_max_. Though our adHOPS method was inspired by localization, we find that it naturally incorporates a dynamic filtering scheme that dramatically improves the scaling of the auxiliary basis with *k*_max_ even when the exciton is fully delocalized. Fig. 4a compares the full (black) and adaptive (green) HOPS dynamics with increasing coupling (*V*). By *V* = 5*λ* the oscillations in the site 3 population report a wave function that is coherently oscillating across 5 sites. Fig. 4b shows the corresponding size of the auxiliary basis as a function of *k*_max_. In all cases the adaptive auxiliary basis (green line) increases much more slowly than the full auxiliary basis (black line).

**Fig. 5:**
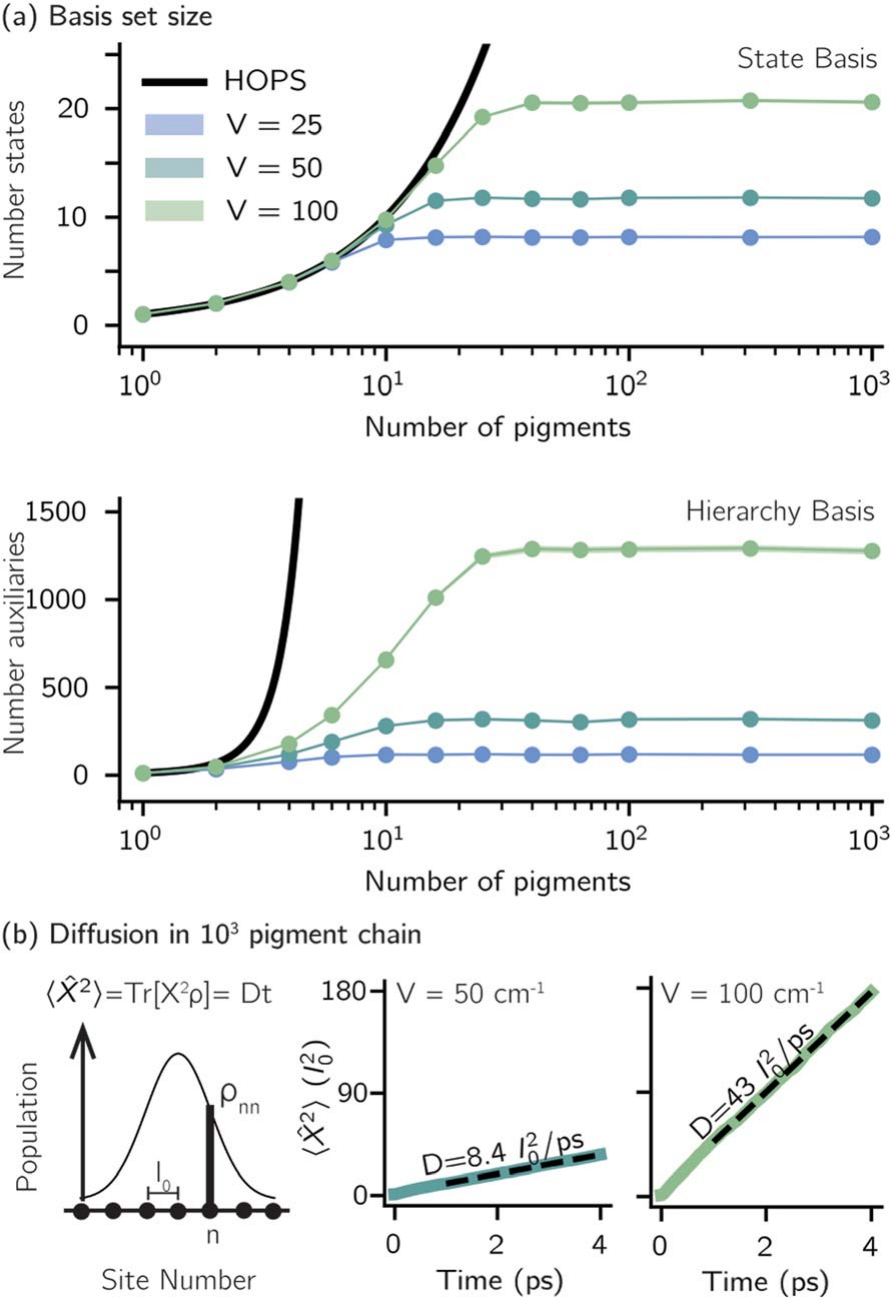
Advantageous scaling of adHOPS simulations for large numbers of pigments. (a) Average number of elements in the adaptive system (top) and auxiliary (bottom) basis for linear chains of different lengths. (b) Exciton diffusion coefficient (in units of molecular spacing, *l*_0_) for a 10^3^ pigment chain from a linear fit to the mean-squared displacement of an excitation starting on the middle pigment 
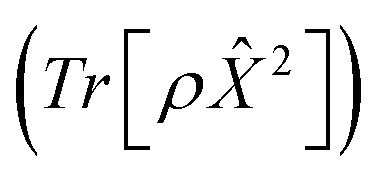
. Parameters: *λ* = *γ* = 50 cm^−1^, *T* = 295 K, *k*_max_ = 10, and *δ* = 3 × 10^−4^. For *V* = 25 and 50 cm^−1^, *N*_traj_ = 10^3^; for *V* = 100 cm^−1^, *N*_traj_ = 5 × 10^3^.

Another perpetual challenge for formally exact methods is their intractable computational scaling with the number of molecules. In HOPS calculations this arises from the scaling of the auxiliary basis. Fig. 5a compares the full (black line) size of the state (top) and auxiliary (bottom) basis to the average size of the adaptive basis (colored lines) as a function of the number of molecules in a linear chain. Compared to the full auxiliary basis, the size of the adaptive auxiliary basis scales favorably with respect to the number of molecules in the aggregate. Moreover, both the auxiliary and state bases in adHOPS calculations show a plateau beyond a threshold size of the linear chain (*N*_pig_ > N*), indicating the onset of size invariant scaling. In the ESI,† we compare the CPU time required for full and adaptive HOPS calculations (*V* = 50 cm^−1^). We find that adaptive calculations are faster than full calculations starting around *N*_pig_ = 10, and we also demonstrate the onset of size invariance (*i.e.*, 
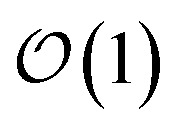
) scaling of CPU time for large aggregates. In other words, increasing the number of pigments beyond a threshold size does not increase the computational expense of an adHOPS calculation. Thus, for localized excitons, the size invariance of adHOPS allows for calculations on scales that were previously unachievable for formally exact methods.

Our adaptive HOPS algorithm offers a computationally tractable approach for formally exact calculations of mesoscale quantum dynamics. As a proof-of-concept, we demonstrate the ability to simulate exciton diffusion on a linear chain of 10^3^ molecules within the formally exact framework of adHOPS (Fig. 5b). Exciton diffusion is a common experimental observable extracted from non-linear microscopies^2^ but is challenging to simulate on long length scales.^35–37^ Using adHOPS, simulating exciton diffusion in a linear chain of 10^3^ pigments is computationally tractable because for *V* = 100 cm^−1^ it requires, on average, less than 2 × 10^3^ auxiliary wave functions and 20 pigment states. The corresponding HOPS simulation would require an auxiliary basis containing more than 10^23^ auxiliary wave functions.

## Conclusions

To summarize, our adaptive HOPS (adHOPS) algorithm:

1. Is a formally exact solution to the time-evolution of a quantum state coupled to a non-Markovian thermal reservoir,

2. Is embarrassingly (or ‘perfectly’) parallel,^24^ and

3. Achieves size-invariant (*i.e.*, 
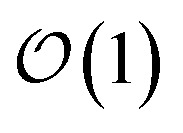
) scaling for molecular aggregates that are substantially larger than the exciton delocalization extent in the material.

This combination of properties allows us to perform non-perturbative, non-Markovian simulations involving an arbitrary number of pigments in physically relevant parameter regimes, thus laying the foundation for mesoscale quantum dynamics simulations of excited-state carriers in molecular materials. Currently, our adaptive algorithm assumes that each pigment has an independent thermal environment composed of overdamped vibrations, but future developments will allow for a broader class of mechanisms involving high-frequency intra-molecular vibrations^38–40^ and Peierls-type electron-vibration coupling.^3^ Looking forward, we believe that adHOPS provides a promising new direction for simulations of a broad range of organic semiconductors including photosynthetic membranes,^16,41^ molecular thin films,^42,43^ and organic photovoltaic heterojunctions.^17–19,44^

## Data availability

The data that supports the findings of this study, the scripts used to run calculations, and the code required to generate the figures are available at DOI: 10.5281/zenodo.4597068. The most recent release of MesoHOPS is available through GitHub at https://github.com/MesoscienceLab/mesohops. The source code used for these calculations is available at DOI: 10.5281/zenodo.4592583.

## Author contributions

Investigation, software, writing – LV, JKL, DIGB; formal analysis, visualization – LV, JKL; conceptualization, funding acquisition, supervision – DIGB.

## Conflicts of interest

There are no conflicts to declare.

## Note added after first publication

This version replaces the manuscript published on 17th June 2021 which contained errors in the application of proof corrections impacting several of the mathematical equations. The RSC apologises for any confusion.

## Supplementary Material

SC-012-D1SC01448J-s001
