# Supplementary material for: Formally exact simulations of mesoscale exciton dynamics in molecular materials
Source: Chem Sci. 2021 May 31;12(28):9704–11. doi: 10.1039/d1sc01448j (PMC8293828; doi:10.1039/d1sc01448j)
Supplement: SC-012-D1SC01448J-s001 [file SC-012-D1SC01448J-s001.pdf]

Electronic Supplementary Material (ESI) for Chemical Science.
This journal is © The Royal Society of Chemistry 2021

Supplementary Information: Formally exact simulations of mesoscale exciton dynamics in molecular materials

Leonel Varvelo, Jacob K. Lynd, and Doran I. G. Bennett*
Department of Chemistry, Southern Methodist University, PO Box 750314, Dallas, TX, USA

I. ADAPTIVE BASIS ALGORITHM

Below we describe the adaptive algorithm for the normalized non-linear HOPS equation

$$\begin{aligned}\hbar\partial_t|\psi_t^{(\vec{k})}\rangle = \big(-i\hat{H}_S - \vec{k}\cdot\vec{\gamma} - \Gamma_t + \sum_n \hat{L}_n(z^*_{n,t}+\xi_{n,t})\big)|\psi_t^{(\vec{k})}\rangle \\ + \sum_n \vec{k}[n]\gamma_n\hat{L}_n|\psi_t^{(\vec{k}-\vec{e}_n)}\rangle \\ - \sum_n (\frac{g_n}{\gamma_n})(\hat{L}_n^\dagger - \langle\hat{L}_n^\dagger\rangle_t)|\psi_t^{(\vec{k}+\vec{e}_n)}\rangle,\end{aligned} \tag{1}$$

where

$$\begin{aligned}\Gamma_t = \sum_n \langle\hat{L}_n\rangle_t \,\mathrm{Re}[z^*_{n,t}+\xi_{n,t}] - \sum_n(\frac{g_n}{\gamma_n})\mathrm{Re}[\langle\psi_t^{(\vec{0})}|\hat{L}_n^\dagger|\psi_t^{(\vec{e}_n)}\rangle] \\ + \sum_n(\frac{g_n}{\gamma_n})\,\langle\hat{L}_n^\dagger\rangle_t\,\mathrm{Re}[\langle\psi_t^{(\vec{0})}|\psi_t^{(\vec{e}_n)}\rangle]\end{aligned} \tag{2}$$

ensures normalization of the physical wave function. We define the derivative error in terms of Euclidean distance between the true derivative vector and the effective derivative vector constructed using the adaptive basis. The key equations (below) provide an upper bound on the derivative error squared and are derived by considering all possible flux contributions in the normalized non-linear HOPS equation (eq. (1)), excluding higher order effects introduced through the normalization correction (Γ_t).

A. Basis Sets

The adaptive basis at the previous time point (t) is defined as the direct sum of a truncated auxiliary and state basis ($\mathbb{B}_t = \mathbb{A}_t \bigoplus \mathbb{S}_t$). In practice, this means that when a state n is not in the adaptive state basis at the previous time point ($n \notin \mathbb{S}_t$) the coefficient of that state is necessarily zero for all auxiliary wave functions at that time point (and vice-versa for an auxiliary $\vec{k} \notin \mathbb{A}_t$). In the following, we will refer to auxiliaries ($\vec{k} \in \mathbb{A}_t$) and states ($n \in \mathbb{S}_t$) that belong to the adaptive basis at the previous time point as 'populated' because they are the only elements with non-zero coefficients.

Given an adaptive basis at the previous time point, the challenge of constructing the new adaptive basis can be split into two pieces: constructing the new auxiliary basis ($\mathbb{A}_{\mathrm{new}}$) and the new state basis ($\mathbb{S}_{\mathrm{new}}$). For each of these pieces, we must determine which populated elements will remain in the new basis ($\mathbb{A}_p, \mathbb{S}_p$) and which 'boundary elements' that were not in the previous basis need to be included ($\mathbb{A}_b, \mathbb{S}_b$). The new adaptive basis is $\mathbb{B}_{t+\Delta t} = \mathbb{A}_{\mathrm{new}} \bigoplus \mathbb{S}_{\mathrm{new}}$, where $\mathbb{A}_{\mathrm{new}} = \mathbb{A}_p \cup \mathbb{A}_b$ and $\mathbb{S}_{\mathrm{new}} = \mathbb{S}_p \cup \mathbb{S}_b$. At each step, we ensure that the error introduced by truncating the basis set is bounded below an error threshold $\delta_{A/S,p/b}$ such that $\delta^2 = \delta^2_{A,p} + \delta^2_{A,b} + \delta^2_{S,p} + \delta^2_{S,b}$. Because the total basis is constructed as the direct sum of the auxiliary and state bases, there is a coupling between the construction of the new auxiliary basis and the new state basis. As a result, the order of basis set construction can influence the results. Though these operations can be performed in any order, in the equations below we assume that the new auxiliary basis is constructed before the new state basis.

For clarity, we assume there is a single thermal environment associated with each molecule. The equations we provide have straightforward extensions for the case of multiple thermal environments on each molecule.

* doranb@smu.edu

B. Flux components

In the following, we will consider how different flux components contribute to the error when basis set elements are neglected in the adaptive basis. To simplify our presentation, we begin by decomposing the components of eq. (1) into convenient pieces. In the following, we revert to a vector notation for the auxiliary wave functions where $\psi_t^{(\vec{k})}[n]$ is the coefficient of the nth state on the $\vec{k}$ auxiliary wave function.

It is convenient to group fluxes that connect populated basis set elements

$$D^{(\vec{k},\vec{k}')}[n,n'] = \hat{K}_{\vec{k},n\leftarrow\vec{k}',n'}(t)\psi_t^{(\vec{k}')}[n'] \tag{3}$$

where $\hat{K}_{\vec{k},n\leftarrow\vec{k}',n'}(t)$ is the time-evolution operator for the complete hierarchy constructed using the adaptive basis for the previous time point ($\mathbb{B}_t$). We will make use of the fact that

$$\partial_t \psi_t^{(\vec{k})}[n] = \sum_{(\vec{k}',n')\in\mathbb{B}_t} D^{(\vec{k},\vec{k}')}[n,n'] \tag{4}$$

as long as $(\vec{k},n) \in \mathbb{B}_t$.

We decompose the different flux contributions to elements outside the populated basis into three basic groups. First, those fluxes that change the state index

$$F^{(\vec{k})}[m,n] = -i\hat{H}_S[m,n]\psi_t^{(\vec{k})}[n]/\hbar \tag{5}$$

arise from the system Hamiltonian inducing coupling between states within a single auxiliary (first line, eq. (1)). Second, those fluxes that increase the auxiliary index ($\vec{k}+\vec{e}_n \leftarrow \vec{k}$)

$$I_+^{\vec{k}}[n] = \gamma_n(\vec{k}[n]+1)\psi_t^{(\vec{k})}[n]/\hbar \tag{6}$$

arise from the second line of eq. (1). Third, those fluxes that can decrease the auxiliary index ($\vec{k}-\vec{e}_n \leftarrow \vec{k}$) which we divide into

$$I_-^{\vec{k}}[n] = -\frac{g_n}{\gamma_n}\psi_t^{(\vec{k})}[n]/\hbar \tag{7}$$

arising from the $\hat{L}_n$ in the third line of eq. (1) and

$$G_{-,n}^{\vec{k}}[m] = \frac{g_n}{\gamma_n}\langle\hat{L}_n\rangle_t\,\psi_t^{(\vec{k})}[m]/\hbar \tag{8}$$

where the $\langle\hat{L}_n\rangle_t$ term allows flux from the m state coefficients on the $\vec{k}$ auxiliary to the m state coefficients on the $\vec{k}-\vec{e}_n$ auxiliary.

C. Auxiliary Basis: Populated Wave Functions

The first step in constructing the new auxiliary basis is to determine which of the populated auxiliary wave functions ($\vec{k} \in \mathbb{A}_t$) can be neglected while ensuring the associated derivative error is below the threshold $\delta_{A,p}$. To determine the error associated with neglecting one populated auxiliary wave function, we consider all of its possible contributions to the derivative vector.

The simplest contribution is the derivative of the coefficients for each populated state in the auxiliary vector. Using the sum property defined in eq. (4) we can write this squared error term as

$$\begin{aligned}&\sum_{n\in\mathbb{S}_t}|\sum_{(\vec{k}',n')\in\mathbb{B}_t} D^{(\vec{k},\vec{k}')}[n,n']|^2\\ &= ||\partial_{t,\mathbb{B}_t}|\psi_t^{(\vec{k})}\rangle||^2\end{aligned} \tag{9}$$

where in the second line we have written $\partial_{t,\mathbb{B}_t}$ to remind us that this equation is an abridgement that only holds for the populated states of the $\vec{k}$ auxiliary wave function (i.e., the components of the auxiliary wave function that are in the adaptive basis at the previous time point).

In addition to its own derivative components, the populated auxiliary $\vec{k}$ can also contribute to the derivative by providing flux. We avoid double-counting error terms included in eq. (9) by only considering non-populated states ($m \notin \mathbb{S}_t$) in the squared error term associated with the $\vec{k} \leftarrow \vec{k}$ flux

$$\begin{aligned} &\sum_{m \notin \mathbb{S}_t} |\sum_{n \in \mathbb{S}_t} F^{(\vec{k})}[m,n]|^2 \\ &= ||(\hat{H} - \hat{P}_{\mathbb{S}_t}\hat{H}\hat{P}_{\mathbb{S}_t})|\psi_t^{(\vec{k})}\rangle||^2/\hbar^2 \end{aligned} \tag{10}$$

where the second line is a convenient operator expression for these terms making use of $\hat{P}_{\mathbb{S}_t}$, the operator that projects onto the populated states ($\mathbb{S}_t$). The squared error arising from the flux towards auxiliaries with a larger index is given by

$$\sum_{n \in \mathbb{S}_t} |I_+^{\vec{k}}[n]|^2 \Theta[\vec{k} + \vec{e}_n, \mathbb{A}] \tag{11}$$

where

$$\Theta[\vec{k}, \mathbb{A}] = \begin{cases} 1 & \text{if } \vec{k} \in \mathbb{A} \\ 0 & \text{otherwise} \end{cases} \tag{12}$$

ensures we only consider flux terms that lead to legal members of the auxiliary basis (since all others should be neglected). The squared error associated with the flux towards auxiliaries with a smaller index is given by

$$\begin{aligned} &\sum_{n \in \mathbb{S}_t} (|I_-^{\vec{k}}[n] + G_{-,n}^{\vec{k}}[n]|^2 + \sum_{n \neq m \in \mathbb{S}_t} |G_{-,n}^{\vec{k}}[m]|^2)\Theta[\vec{k} - \vec{e}_n, \mathbb{A}] \\ &\leq \sum_{n \in \mathbb{S}_t} \Theta[\vec{k} - \vec{e}_n, \mathbb{A}] \left|\frac{g_n}{\gamma_n}\right|^2 (|\psi_t^{(\vec{k})}[n]|^2 + \langle \hat{L}_n^\dagger \rangle_t^2 ||\psi_t^{(\vec{k})}||^2)/\hbar^2 \end{aligned} \tag{13}$$

where we have rearranged terms in the second line to generate a more convenient expression, at the price of introducing an upper bound.

If we neglect the $\vec{k}$ auxiliary wave function, then by the end of the next time step the coefficients are forced to zero. This implicitly introduces a fictitious derivative constructed to precisely cancel the current amplitude in a single time step. Since this flux does not arise in the HOPS equation, this is an additional squared error term in our derivative

$$||\psi_t^{(\vec{k})}||^2/\Delta t^2 \tag{14}$$

which depends on the simulation time step (Δt).

The square of the derivative error introduced by removing the $\vec{k}$ populated auxiliary wave function ($E_p^2[\vec{k}]$) is bounded by the sum of eqs. (9), (10), (11), (13), and (14). We determine the largest set of auxiliaries that can be removed at the current time point while maintaining the bound $\delta_{A,p}$ on the derivative error. The remaining auxiliaries – those that were in the adaptive basis in the previous time point and will be in the adaptive basis in the next time point – define the set $\mathbb{A}_p$. We note that our selection criterion (maximum number of auxiliaries removed), like all subsequent basis set selections, is not unique and a variety of different algorithms can be used to determine which auxiliaries to keep at each time point while satisfying the error bound.

D. Auxiliary Basis: Boundary Wave Functions

Boundary wave functions – auxiliary wave functions that are members of the full auxiliary basis but were not in the adaptive basis at the previous time point ($\vec{k} \in \mathbb{A} \setminus \mathbb{A}_t$) – have no amplitude to contribute to flux but may still be important to the overall dynamics by accepting amplitude from populated auxiliaries. Naively, one might attempt to calculate the error for neglecting each possible boundary auxiliary $\vec{k} \in \mathbb{A} \setminus \mathbb{A}_t$ which would scale with the size of the full auxiliary basis and be unmanageable for even moderately sized pigment aggregates. However, the only way

for an auxiliary $\vec{k}$ to belong to this set is for it to be connected to one (or more) populated auxiliaries. As a result, it is more efficient to determine the important connections with populated auxiliaries than to directly search for the important boundary auxiliary wave functions.

We can determine an upper bound on the squared error for neglecting boundary auxiliary wave functions in terms of the populated auxiliary $\vec{k}' \in \mathbb{A}_p$ that creates the flux and the mode (n) along which it is connected to the boundary, either from below $(\vec{k}', n, +)$:

$$\big(\Theta[\vec{k}' + \vec{e}_n, \mathbb{A} \setminus \mathbb{A}_t] |I_+^{\vec{k}'}[n]|^2\big), \tag{15}$$

or from above $(\vec{k}', n, -)$:

$$\Theta[\vec{k}' - \vec{e}_n, \mathbb{A} \setminus \mathbb{A}_t] \Big|\frac{g_n}{\gamma_n}\Big|^2 (|\psi_t^{(\vec{k}')}[n]|^2 + \langle \hat{L}_n^\dagger \rangle_t^2 ||\psi_t^{(\vec{k}')}||^2)/\hbar^2 \tag{16}$$

where the second expression arises from the same considerations leading to eq. (13) and is an upper bound. We introduce $\Theta[\vec{k}' \pm \vec{e}_n, \mathbb{A} \setminus \mathbb{A}_t]$ operators to ensure that the each flux term goes to an auxiliary wave function that was not in the adaptive basis at the previous time point.

Treating each of these error terms independently, we construct the largest set of tuples that can be removed $\{(\vec{k}', n, \pm), ...\}$ such that the associated error is less than $\delta_{A,b}$. The set $\mathbb{A}_b$ is composed of all auxiliaries constructed from the remaining tuples $(\vec{k} = \vec{k}' \pm \vec{e}_n)$. This algorithm does not guarantee that the minimal error is achieved since we do not determine which auxiliary each flux term leads to until after the truncation. However, it has the advantage of introducing only a small additional computational cost since the vast majority of all connections to the boundary are negligible due to localization in the auxiliary wave functions.

E. State Basis: Populated States

To strengthen the analogy between the auxiliary and state bases, we introduce a new vector $|\phi_t^{(n)}\rangle$ which contains the coefficient of the nth state across all auxiliaries in the reduced set $\mathbb{A}_p$ (i.e. $\phi_t^{(n)}[\vec{k}] = \psi_t^{(\vec{k})}[n]$ if $\vec{k} \in \mathbb{A}_p$). The construction of the state basis is completely analogous to the auxiliary basis construction. A brief description is provided below for completeness.

For each populated state, we first consider its contribution to the derivative of coefficients for each populated auxiliary wave function

$$\begin{aligned} &\sum_{\vec{k} \in \mathbb{A}_p} | \sum_{(\vec{k}', n') \in \mathbb{A}_p \bigoplus \mathbb{S}_t} D^{(\vec{k}, \vec{k}')}[n, n']|^2 \\ &= ||\hat{P}_{\mathbb{A}_p} \partial_{t, \mathbb{B}_t} (\hat{P}_{\mathbb{A}_p} |\phi_t^{(n)}\rangle)||^2 \end{aligned} \tag{17}$$

where the sums in the first line only considers auxiliary wave function that are in the truncated set of populated wave functions $\mathbb{A}_p$. In the second line, we rewrite that into a convenient operator notation, again recognizing the abridged time-evolution $\partial_{t,\mathbb{B}_t}$ which must be further reduced onto the truncated set of populated auxiliary wave functions by the projection operator $\hat{P}_{\mathbb{A}_p}$.

In addition to their own derivative components, the populates state n can also contribute to the derivative by providing flux. We avoid double-counting error already included in eq. (17) by only considering non-populated states $(m \notin \mathbb{S}_t)$ for the squared error term associated with the $\vec{k} \leftarrow \vec{k}$ flux

$$\begin{aligned} &\sum_{\vec{k} \in \mathbb{A}_p} \sum_{m \notin \mathbb{S}_t} |F^{(\vec{k})}[m, n]|^2 \\ &= V[n] \; ||\phi_t^{(n)}||^2/\hbar^2 \end{aligned} \tag{18}$$

where in the second line we have introduced

$$V[n] = \sum_{m \notin \mathbb{S}_t} |H_s[m, n]|^2 \tag{19}$$

which quantifies the total coupling of state n to all states not included in the previous basis ($m \notin \mathbb{S}_t$). In addition there are the flux terms which can increase the auxiliary index

$$\sum_{\vec{k}\in\mathbb{A}_p} |I_+^{\vec{k}}[n]|^2 \Theta[\vec{k}+\vec{e}_n, \mathbb{A}] \tag{20}$$

or decrease the auxiliary index

$$\sum_{\vec{k}\in\mathbb{A}_p} \Theta[\vec{k}-\vec{e}_n, \mathbb{A}] \Big|\frac{g_n}{\gamma_n}\Big|^2 (|\psi_t^{(\vec{k})}[n]|^2 + \langle \hat{L}_n^\dagger\rangle_t^2 ||\psi_t^{(\vec{k})}||^2)/\hbar^2. \tag{21}$$

Again, eq. (21) represents an upper bound on the squared error.

Finally, the squared derivative error arising from the fictitious flux required to cancel the residual amplitude on the neglected state is given by

$$||\phi_t^{(n)}||^2/\Delta t^2. \tag{22}$$

Using the bound on the squared derivative error given by the sum of eqs. (17), (18), and (20)-(22), we determine the largest set of states ($\{n'\}$) which can be neglected while ensuring the total error is smaller than $\delta_{S,p}$. The set of remaining states we will label $\mathbb{S}_p$.

F. State Basis: Boundary States

The set of states that are not included in the adaptive basis at the previous time point can be important for accurately propagating the time evolution if they accept flux from one (or more) populated states. When the system-bath coupling operators ($\hat{L}_n$) are site-projection operators, the only term in the normalized non-linear HOPS equation which can change the state index is the system Hamiltonian ($\hat{H}_s$), and the corresponding squared error for neglecting the flux into a state $n \in \mathbb{S}\setminus\mathbb{S}_t$ is given by

$$\begin{aligned}\sum_{k\in\mathbb{A}_p} | \sum_{m\in\mathbb{S}_p} F^{(\vec{k})}[n,m]|^2 \\ = \sum_{\vec{k}\in\mathbb{A}_p} |\Psi^{(\vec{k})}[n]|^2/\hbar^2\end{aligned} \tag{23}$$

where

$$|\Psi_t^{(\vec{k})}\rangle = (\hat{H}_s - \hat{P}_{\mathbb{S}_p}\hat{H}_s\hat{P}_{\mathbb{S}_p})\hat{P}_{\mathbb{S}_p}|\psi_t^{(\vec{k})}\rangle \tag{24}$$

provides a convenient operator formulation. Eq. (23) can be further simplified to

$$||\Phi_t^{(n)}||^2/\hbar^2 \tag{25}$$

by defining, in analogy to the definition of $\phi^{(n)}$ above, $\Phi_t^{(n)}[\vec{k}] = \Psi_t^{(\vec{k})}[n]$ where $\vec{k} \in \mathbb{A}_p$ and $n \in \mathbb{S}\setminus\mathbb{S}_t$.

We determine the largest set of states ($n' \in \mathbb{S}\setminus\mathbb{S}_t$) that can be neglected while maintaining $\delta_{S,b}$ as the bound on the derivative error. The remaining states form the set $\mathbb{S}_b$.

G. Hamiltonian couplings

To achieve $\mathcal{O}(1)$ scaling in eqs. (10), (18) and (23), the system Hamiltonian must be sparse. We note that for a physical Hamiltonian that supports coupling over a finite spatial extent (e.g., r^{-3} scaling of dipole-dipole coupling), this sparsity requirement is necessarily fulfilled for large aggregates. The simplest computational approach to leveraging the locality of coupling is to filter the system Hamiltonian so that elements below a threshold (ϵ) are set to zero before the calculation begins.

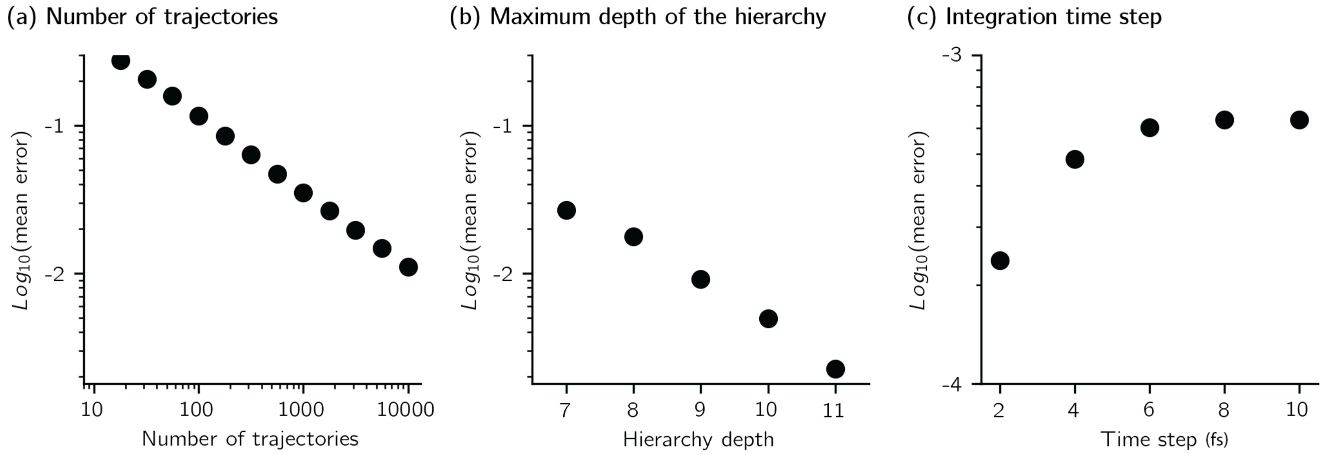

FIG. S1. Comparing error sources in HOPS ensembles. (a) Statistical error from bootstrapping as a function of trajectories with $k_{max} = 10$. (b) Error in population vectors as a function of k_{max} with $N_{\text{traj}} = 10^3$. (c) Error in population vectors as a function of time step with $k_{max} = 10$ and $N_{\text{traj}} = 10^4$. Parameters: V = 50 cm^{-1}, λ = 50 cm^{-1}, γ = 50 cm^{-1}, and T = 295 K.

H. Adaptive Parameters

The adaptive basis at each time point is defined by four error parameters ($\delta_{A,p}, \delta_{A,b}, \delta_{S,p}, \delta_{S,b}$). Instead of specifying each of the four parameters, we select a single parameter δ and, for all calculations presented here, require the error to be equally distributed between the auxiliary and state basis ($\delta_A^2 = \delta_S^2 = \delta^2/2$). The explicit distribution among the sub-parameters is determined at each time point. For the auxiliary basis, the error bound used for the populated auxiliaries is required to obey $\delta_{A,p} \leq \delta_A/\sqrt{2}$. This value is often not saturated, so at each time point we define $\delta_{A,b}^2 = \delta_A^2 - \delta_{A,p}^2$. The equivalent is done for the state basis.

The algorithm above can be partitioned to allow for either the state or auxiliary basis to be treated adaptively while the other is statically defined. For small aggregates, in particular, it is often convenient to not solve for an adaptive state basis since most (or even all) states will be in the adaptive basis most of the time.

II. SOURCES OF ERROR

In a HOPS simulation, there are three sources of error for an ensemble averaged property: the statistical error, associated with a finite number of trajectories, the hierarchy error arising from a truncation of the hierarchy, and the time-step error that arises from numerical integration (in this case fourth-order Runge-Kutta).

Here, we characterize the distance between the outcome of two calculations in terms of the mean norm of the population differences on each pigment

$$\sigma = \frac{1}{N_t} \sum_t \sqrt{\frac{1}{2} \sum_n |\vec{P}_n(t) - \vec{p}_n(t)|^2} \tag{26}$$

where the factor of $\frac{1}{2}$ ensures σ is bounded between 0 and 1.

A. Number of Trajectories

We estimate the statistical error arising from a finite number of trajectories by bootstrapping [1]. We first calculate 10^4 trajectories. Then, for each value of N_{traj} we construct 10^4 ensembles by sampling (with replacement) N_{traj} trajectories from the original ensemble of 10^4 trajectories. We use the half-width of the 95% confidence interval to characterize the expected error arising from finite sampling. Specifically, we construct a vector ($\vec{\Sigma}_t$) with half-width of the 95% confidence interval for each site population at a given time (t) and estimate the statistical error (ϵ)

$$\epsilon = \frac{1}{\sqrt{2} N_t} \sum_t \left\| \vec{\Sigma}_t \right\| \tag{27}$$

where the factor of $\sqrt{2}$ ensures consistency with the error definition. Fig. S1a shows the statistical error as a function of number of trajectories.

B. Depth of the Hierarchy

HOPS calculations are exact for an infinite hierarchy, but numerical calculations require the calculation to consider only auxiliary wave functions up to some maximum depth (k_{max}). To isolate the effects of k_{max} on the population vectors, we minimize the influence of statistical error by comparing simulations at different hierarchy depths using the same noise trajectories. We calculate the error at different depths of the hierarchy by comparing the ensemble average population vector to the results when $k_{max} = 12$, using eq. (26). Fig. S1b shows the mean error vs hierarchy depth for 10^3 trajectories. By $k_{max} = 10$ the hierarchy error is substantially below 0.01, and we use this value of k_{max} for most of our 5 site calculations.

C. Time Step

In numerical integration, the time step affects both the accuracy of the trajectory and the computational cost. To test convergence with respect to the time step (Δt), we consider matched HOPS trajectories with varying Δt values. Figure S1c presents the distribution of mean error (eq. 26) as a function of increasing Δt when compared to population vectors calculated using a time step of 1 fs. The error gradually increases as the time step becomes larger. We did not explore the origin of the integration error, though it is notable that the Markovian mode introduces a timescale of decay that is approximately 10 fs. Except where noted otherwise, for the calculations presented here, we have used a time step of 4 fs which is sufficiently short to sample the Markovian dynamics.

D. Distribution of adaptive error

With the use of an adaptive basis, adHOPS introduces a new form of error into the HOPS framework, arising due to the finite accuracy with which the derivative is calculated at any given time. We calculate the adaptive error for each trajectory in a 10^4 member ensemble as the mean difference (σ, eq. (26)) between the equivalent adHOPS and HOPS trajectories. The distribution of adaptive errors (Fig. S2) shows errors spreading more than two orders of magnitude. To explore how error evolves in individual trajectories, we plot the error vs time for trajectories that show extremely high error (red box, red line), moderately high error (green box, green lines), and 'nominal' error behavior (blue box, blue lines). The highest error arises from adHOPS trajectories that diverge from the equivalent HOPS calculations, though these represent around 0.1% of the ensemble. The moderately high errors arise from adHOPS trajectories that show periods in which they drift away from the corresponding HOPS trajectory before returning, as seen by the peak like structure in the error plots. The 'nominal' error of most adHOPS trajectories show small fluctuations in the vicinity of the corresponding HOPS trajectory. We note that the mean adHOPS ensemble population dynamics are even more similar to the corresponding HOPS calculations than these results suggest, due to cancellation of errors. The black line in Fig. S2 shows the error between the ensemble average adHOPS population trajectory compared to the corresponding HOPS ensemble average and has a mean value of 3×10^{-3}.

III. CONVERGENCE IN ADHOPS

In an adHOPS calculations we have two convergence parameters that define the hierarchy basis used in our calculations: the maximum depth of the hierarchy (k_{max}) and the bound on the derivative error (δ). We explored the possibility of a coupling between these two convergence criteria that would lead to changes in the converged value of one (e.g., k_{max}) for different values of the other parameter (δ). Our approach began with a scan over δ values in logarithmic half-steps (e.g., log(δ) = -1, -1.5, -2, etc.) until the difference between two calculations (σ, eq. (26)) with δ separated by 1 log unit was less than 0.01. We then ran a similar convergence scan for k_{max} with values increasing by steps of 1, until the difference between two calculations (σ, eq. (26)) with k_{max} separated by 2 was less than 0.01. We then ran a new δ convergence scan at the converged value of k_{max}. In all cases we found that this second δ scan returns the same convergence value as the first, suggesting that the δ and k_{max} scans are, at least to a first approximation, independent.

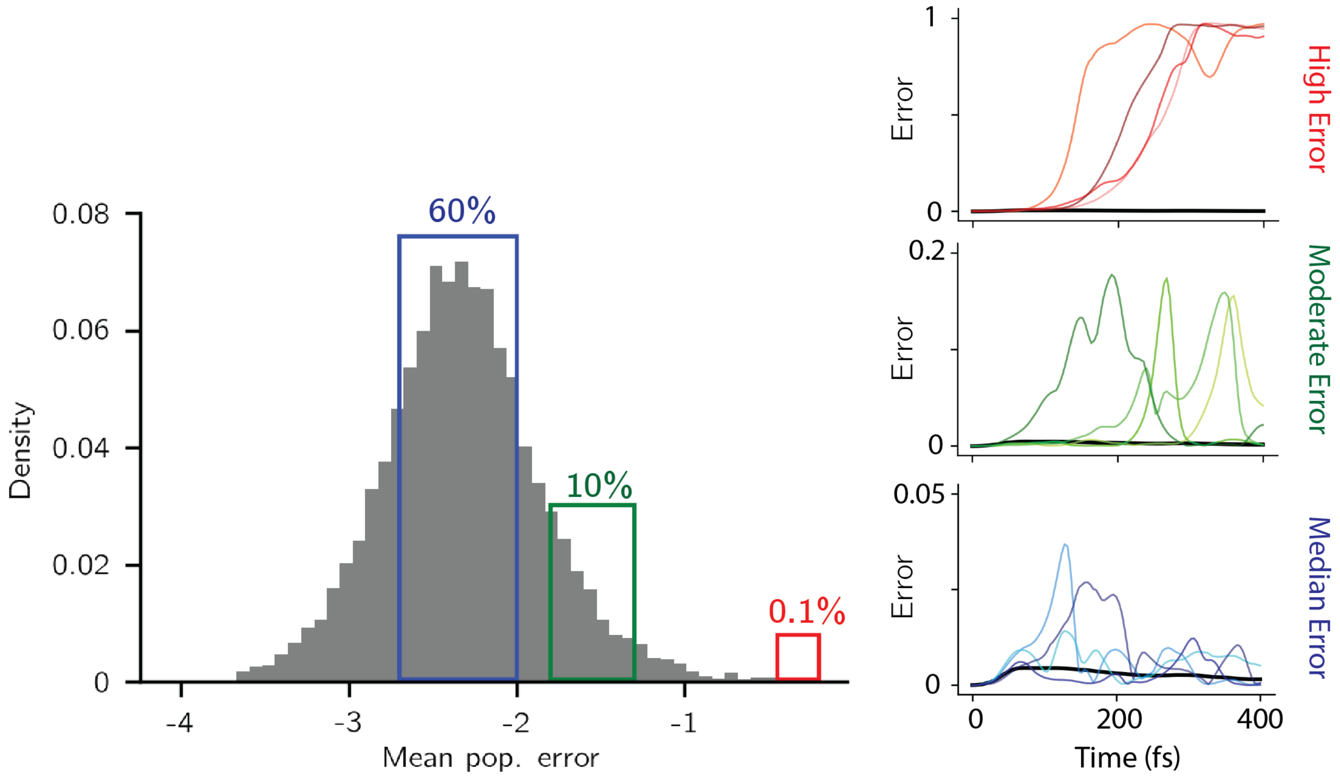

FIG. S2. Population error of individual trajectories and their ensemble distribution. The ensemble contains 10^4 matched HOPS and adHOPS ($\delta = 10^{-3}$) trajectories. Four trajectories each from median (blue lines), moderate (green lines), and high-error (red lines) subsets of the ensemble are plotted with the ensemble mean (black line). Parameters: V = 50 cm^{-1}, $\lambda = 50$ cm^{-1}, $\gamma = 50$ cm^{-1}, T = 295 K, $k_{max} = 10$, $\delta = 10^{-3}$, $N_{\rm traj} = 10^4$, and Δt = 4 fs.

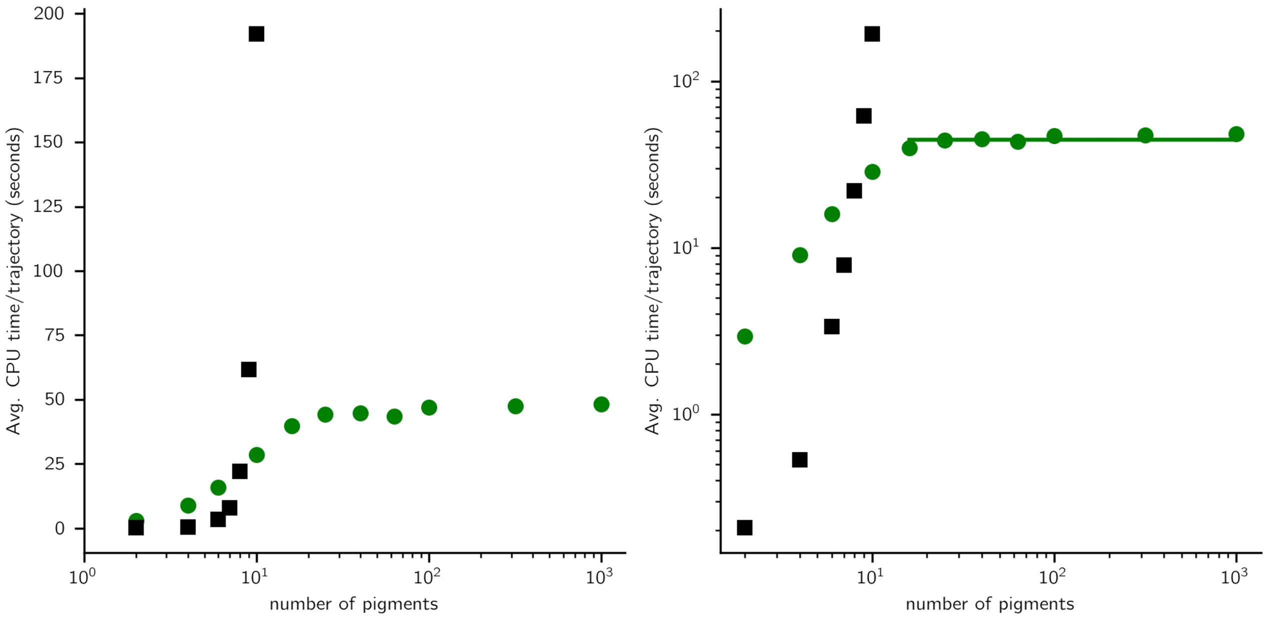

FIG. S3. Average CPU time for running HOPS (black squares) and adHOPS (green circle) simulations. The green line in the right panel shows a linear fit to the data from $N_{pig} = 16$ to 1000, with a coefficient of 0.0277. Calculations were run using Intel Xeon 2.1 GHz processor. Parameters: $V = 50$ cm^{-1}, $\lambda = 50$ cm^{-1}, $\gamma = 50$ cm^{-1}, $T = 295$ K, $k_{max} = 10$, $\delta = 3 \times 10^{-4}$, and Δt = 4 fs.

IV. CPU TIMING

In Fig. S3, we compare the CPU time required to run matched HOPS (black squares) and adHOPS (green circles) calculations for the sequence of linear chains presented in Fig. 6 of the main text. We measure the CPU time as the time required to propagate the equation-of-motion, excluding any time spent in the initial setup. HOPS calculations were only performed for $N_{pig} \leq 10$ due to the extreme computational expense for calculations of larger chains. We find that starting at $N_{pig} = 9$, adHOPS calculations require less CPU time per trajectory than the matched HOPS calculations, and adHOPS calculations including up to 10^3 pigments (and beyond) can be performed conveniently.

Using the log-log plot for CPU time vs system size (Fig. S3, right panel) we determine a residual scaling of $\mathcal{O}(N_{pig}^{0.03})$ for the adHOPS calculations in the size-invariant region. This results in a 20% increase in CPU time per trajectory when increasing the linear chain length from $N_{pig} = 16$ to 1000. We have not yet identified the origin of the residual scaling.

V. CODE

With the exception of the SI section on CPU timing (above), all calculations presented here used the version 1.00 of MesoHOPS. The CPU timing section used version 1.10 of MesoHOPS, which is also the version archived with Zenodo. The changes in the code do not influence any conclusions though numerical differences may be observed.

MesoHOPS V1.1.0: http://doi.org/10.5281/zenodo.4592583

MesoHOPS V1.0.0: (https://github.com/MesoscienceLab/mesohops, commit: 86e991917c9e57d63dfc9b5e140d1f6112544f98)

REFERENCES

[1] A. C. Davison and D. V. Hinkley. *Bootstrap Methods and their Application*. Cambridge Series in Statistical and Probabilistic Mathematics. Cambridge: Cambridge University Press, 1997. ISBN: 978-0-521-57471-6. DOI: 10.1017/CBO9780511802843. URL: https://www.cambridge.org/core/books/bootstrap-methods-and-their-application/ED2FD043579F27952363566DC09CBD6A.
